# Inter- and intra-study reproducibility of LV volume analysis performed by technologists

**DOI:** 10.1186/1532-429X-14-S1-T11

**Published:** 2012-02-01

**Authors:** Chris Lawton, Cornelius Imobeke, Elisa McAlindon, Jessica Harris, Chiara Bucciarelli-Ducci

**Affiliations:** 1 Bristol Heart Institute, NIHR Cardiovascular BRU, Bristol, UK

## Background

Cardiac magnetic resonance (CMR) imaging studies are increasingly being carried out in patients with cardiovascular disease. In a high throughput centre (>1,200 scans per year) technologists can potentially provide significant help in assisting with LV volumes analysis but the inter- and intra-observer variability of this task has not been reported.

## Methods

Two CMR technologists with no previous experience in analysing LV volumes assessed 20 CMR studies in ischemic heart disease patients. 10 studies were re-analysed 24 hours after the first analysis.

Volumes and mass were analysed using semi-automated software (Argus, Siemens) following a 2hr tutorial on how to use the software.

## Results

Intra-observer variability was assessed using intraclass correlation coefficient (ICC); inter-observer variability was assessed using Bland Altman plots for agreement. Intra-observer variability was low for both observers (ICC; observer 1 EDV: 0.99, ESV: 0.93, mass: 0.94 vs observer 2 EDV: 0.97, ESV: 0.97, mass: 0.94). Inter-observer variability was lowest for volumes and highest for mass (Table 1).

**Table 1:** Inter-observer variability

Observer		EDV (ml)	ESV* (ml)	mass (g)
1 vs 2	Mean (SD) difference	2.4 (6.1)	0.3 (0.2)	5.1 (11.3)
1 vs 2	Mean (SD)	163.4 (30.3)	4.4 (0.4)	147.8 (27.0)
1 vs 2	Coefficient of variation (%)	3.7	4.4	7.6

Summary statistics calculated are mean and SD of differences, mean and SD of values. Differences between observers are assessed using Bland-Altman plots. *log-transformed for skewed distribution.

## Conclusions

Technologists with no previous experience in analysising LV parameters can analyse LV volumes after a short tutorial on a semi-automated software with good reproducibility, and a low inter- observer variability.

## Funding

NIHR Cardiovascular BRU, Bristol Heart Institute.

